# Changes in Frailty and Incident Cancer: Evidence From the Health and Retirement Study

**DOI:** 10.1002/jcsm.70164

**Published:** 2025-12-16

**Authors:** Zhaoting Bu, Xinying Chen, Xiaoyue Liu, Bing Yin, Sanyu Ge, Xin Zheng, Changhong Xu, Hong Zhao, Yi Li, Xiangrui Li, Hanping Shi

**Affiliations:** ^1^ Department of Clinical Nutrition Beijing Shijitan Hospital, Capital Medical University Beijing China; ^2^ Center for Clinical Nutrition and Department of Colorectal Surgery, The First Affiliated Hospital of Wenzhou Medical University Wenzhou Zhejiang China; ^3^ Key Laboratory of Cancer FSMP for State Market Regulation Beijing China; ^4^ Department of Clinical Oncology, LKS Faculty of Medicine, the University of Hong Kong Hong Kong China

**Keywords:** cancer, dynamic nature, epidemiology, frailty, the Health and Retirement Study

## Abstract

**Background:**

Although frailty has been identified as a potential risk factor for cancer, most previous studies have only considered frailty status at a single time point. The relationship between dynamic changes in frailty and incident cancer is less well understood. This study aimed to evaluate the associations of both baseline frailty status and changes in frailty status with subsequent cancer risk.

**Methods:**

Data were derived from the Health and Retirement Study (HRS), a nationally representative prospective cohort in the United States. Frailty was assessed using a 29‐item Rockwood frailty index and categorized as robust, pre‐frail or frail. Changes in frailty status were determined over a 2‐year period. Incident cancer was identified through self‐reported physician diagnoses. Cox proportional hazards models were used to estimate hazard ratios (HRs) and 95% confidence intervals (CIs), adjusting for demographic, lifestyle and health‐related covariates.

**Results:**

A total of 11 661 participants (63.1% female; mean age: 67.1 years) were included in the baseline frailty analysis, and 10 178 participants (63.8% female; mean age: 66.3 years) were included in the frailty change analysis. During a median follow‐up of 7.2 years, baseline frailty was associated with a significantly increased risk of incident cancer (frail vs. robust: HR 1.61, 95% CI 1.27–2.02; pre‐frail vs. robust: HR 1.46, 95% CI 1.17–1.83). Over the 2‐year transition period, participants who progressed from robust to pre‐frail/frail status had a higher cancer risk compared to those who remained robust (HR 2.50, 95% CI 1.74–3.61). Conversely, frail individuals who improved to pre‐frail or robust status had a reduced cancer risk relative to those who remained frail (HR 0.66, 95% CI 0.48–0.90). Similar risk reduction was observed among pre‐frail individuals who recovered to robust status (HR 0.51, 95% CI 0.34–0.76). Additionally, greater increases in frailty index over timeremained associated with elevated cancer risk after multivariable adjustment (highest vs. lowest quartile of ΔFI: HR 1.35, 95% CI 1.13–1.63; *p* for trend < 0.001).

**Conclusions:**

Both baseline frailty and changes in frailty status are independently associated with cancer risk. Frailty progression significantly increases the risk of incident cancer, whereas recovery from frailty is associated with reduced risk. These findings underscore the importance of dynamic frailty monitoring and suggest that interventions targeting frailty warrant investigation for potential cancer risk reduction.

## 1 Introduction

With the global population ageing rapidly, frailty has become an increasingly important public health concern [1, 2]. Frailty is a clinical geriatric syndrome characterized by a decline in physiological reserves and increased vulnerability to external stressors, leading to higher risks of adverse health outcomes such as falls, disability, hospitalization and mortality [1, 3]. Compared with working older adults or the broader older population, the retirement phase is marked by distinct lifestyle patterns, healthcare utilization, social engagement, and daily routines, which may translate into different health vulnerabilities. Meanwhile, cancer remains a leading cause of morbidity and mortality worldwide [4, 5]. Although traditionally studied as separate entities, recent research suggests that frailty and cancer may share overlapping biological mechanisms, including chronic inflammation, immune dysfunction, and impaired cellular repair [6, 7]. Understanding the role of frailty in cancer development is essential for identifying individuals at risk individuals and informing preventive strategies.

A growing body of literature has documented the negative impact of frailty on cancer outcomes. Individuals with cancer who are frail are more likely to experience treatment‐related complications, reduced treatment tolerance, prolonged recovery, and poorer overall survival [8, 9, 10]. Frailty also substantially impairs quality of life and increases healthcare burden among patients with cancer [11, 12]. However, most existing research has primarily focused on the prognostic role of frailty after a cancer diagnosis. There is limited evidence regarding whether frailty‐particularly its progression over time‐maycontribute to the development of cancer itself. Considering that frailty is not a static condition and may be reversed or improved through appropriate interventions, studying changes in frailty status could offer a more dynamic and predictive view of cancer risk.

In this study, we used data from the Health and Retirement Study (HRS), a nationally representative longitudinal cohort of older adults in the United States. We aimed to investigate the association between changes in frailty status and the risk of incident cancer. We hypothesized that individuals who progressed to greater frailty would have an increased risk of developing cancer, whereas those who recovered from frailty would have a decreased risk. Our findings may provide novel insights into the role of frailty dynamics in cancer prevention and early identification among ageing populations.

## 2 Methods

### 2.1 Study Design and Population

This study was based on data from the HRS, an ongoing, nationally representative longitudinal survey. Detailed information on the design and methodology of the HRS has been previously published. For this analysis, we defined Wave 10 (2010) as the baseline and Wave 11 (2012) as the follow‐up assessment for evaluating changes in frailty status. Participants were subsequently followed through Wave 14 (2018) to identify incident cancer cases.

**FIGURE 1:**
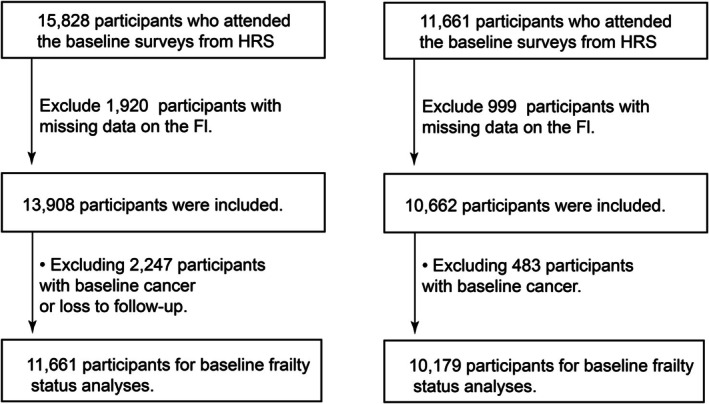
Selection process of the study population.

We first included all participants with available data in Wave 10 of the HRS. Individuals were excluded if they had missing information required to compute the frailty index (FI), had a cancer diagnosis at baseline or were lost to follow‐up. For analyses focusing on frailty progression or recovery, participants without a second frailty assessment at Wave 11 were further excluded. The final analytic sample included individuals with complete frailty data at both time points and without a cancer diagnosis at baseline (Figure 1).

### 2.2 Frailty Assessment

Frailty was assessed using an FI, developed following the deficit accumulation approach. This index was constructed from 29 health variables covering multiple domains, including chronic conditions (excluding cancer), symptoms, functional limitations, cognitive impairment and psychological health. Each item was scored as 0 (no deficit) or 1 (deficit present), except for the cognitive function variable, which was scored continuously between 0 and 1, with higher values indicating worse cognitive performance. The FI for each participant was calculated as the total number of deficits divided by 29, yielding a score ranging from 0 to 1 (Table S1). Based on established thresholds, frailty status was categorized as robust (FI ≤ 0.10), pre‐frail (0.10 < FI < 0.25) or frail (FI ≥ 0.25).

To assess the dynamic nature of frailty, we compared frailty status at Waves 10 and 11. Participants were classified into one of three frailty trajectory groups: robust, pre‐frail and frail. We also calculated the absolute change in FI (Wave 11 FI minus Wave 10 FI) as continuous measures to capture frailty dynamics.

### 2.3 Outcome Ascertainment

The primary outcome of this study was incident cancer, determined through self‐reported physician diagnosis. At each wave, participants were asked whether a doctor had ever told them they had cancer or a malignant tumour, excluding minor skin cancers. Incident cancer was defined as a positive report occurring after the baseline or second frailty assessment, depending on the analysis.

For baseline frailty status analyses, follow‐up began in Wave 10 (2010), whereas for frailty change analyses, follow‐up started in Wave 11 (2012). Participants were followed until the first report of cancer, death or the most recent wave they completed, whichever occurred first. The censoring date was defined as the date of the last completed interview, with Wave 14 (2018) as the latest available wave. Death status was tracked through the HRS follow‐up records.

### 2.4 Covariates

Potential confounding variables included demographic, behavioural and biological factors. These comprised age, sex, physical activity (PA) (had/no, mild active, moderately active, vigorously active), education level (categorized as less than high school, high school graduate or equivalent, some college or college graduate), marital status (married, others(separated/divorced/widowed/never married)), alcohol use (yes/no), smoker status (yes/no) and self‐reported hypertension (yes/no).

### 2.5 Statistical Analysis

For descriptive statistics, continuous variables were expressed as mean [standard deviation (SD)] or median [interquartile range (IQR)], and categorical variables as number (percentage). Cox proportional hazards models were used to estimate the association between frailty status (and changes in frailty status) and the risk of incident cancer. Hazard ratios (HRs) and 95% confidence intervals (CIs) were calculated after adjustment for the aforementioned covariates. In sensitivity analyses, we employed Fine and Gray's competing risk models to account for the possibility that death could preclude cancer diagnosis. Given ongoing debate regarding FI cut‐offs for defining frailty categories, we conducted sensitivity analyses using two alternative commonly used cut‐offs: (cut‐off value 1) frail: FI ≥ 0.21, pre‐frail: 0.10 < FI < 0.21, robust: FI ≤ 0.10; and (cut‐off value 2) frail: FI ≥ 0.25, pre‐frail: 0.08 < FI < 0.25, robust: FI ≤ 0.08. To assess the robustness of the FI construction, we additionally built an expanded 31‐item FI by incorporating smoking and alcohol use items and repeated the main analyses using this index. To evaluate whether changes in frailty were associated with cancer risk independent of baseline frailty, we repeated analyses of changes in frailty status with further adjustment for baseline FI/changes in frailty status. To minimize potential reverse causality, we repeated the main analyses after excluding participants who developed cancer within the first 3 months of follow‐up. Stratified analyses by sex and age group (< 65 vs. ≥ 65 years) were also conducted. Statistical interactions were tested using likelihood ratio tests comparing models with and without interaction terms. All statistical analyses were carried out by R software (Version 4.1.2). All *p* values were two‐sided, and *p* < 0.05 was considered statistically significant.

## 3 Results

### 3.1 Participant Characteristics and Follow‐Up

A total of 11 661 participants (63.1% female; mean age 67.1 years) from the HRS cohort were included in the analysis of baseline frailty status. As summarized in Table 1, individuals classified as frail at baseline tended to be older, more likely to be female, less likely to be married and had lower education levels and lower physical activity. For the analysis of longitudinal changes in frailty status, 10 178 participants (63.8% female; mean age 66.3 years) were included based on data availability. Their baseline characteristics are shown in Table 2. The median follow‐up was 7.2 years, during which 2151 participants died. In the frailty change analysis, the median follow‐up duration was 6.0 years, with 1547 deaths recorded during this period.

**TABLE 1:** Baseline (Wave 10) characteristics of participants for baseline frailty status analyses.

		Overall	Robust	Pre‐frail	Frail	p
*n*	Level	11 661	1396	5175	5090	
Gender (%)	Male	4296 (36.84)	571 (40.90)	1947 (37.62)	1778 (34.93)	< 0.001
	Female	7365 (63.16)	825 (59.10)	3228 (62.38)	3312 (65.07)	
Age (years) (mean [SD])		67.05 (11.98)	61.77 (11.03)	66.45 (11.20)	69.12 (12.47)	< 0.001
Physical activity						
Vigorous (%)	No	9548 (81.88)	960 (68.77)	3981 (76.93)	4607 (90.51)	< 0.001
	Had	2113 (18.12)	436 (31.23)	1194 (23.07)	483 (9.49)	
Moderate (%)	No	7106 (60.94)	564 (40.40)	2776 (53.64)	3766 (73.99)	< 0.001
	Had	4555 (39.06)	832 (59.60)	2399 (46.36)	1324 (26.01)	
Mild (%)	No	6046 (51.85)	525 (37.61)	2205 (42.61)	3316 (65.15)	< 0.001
	Had	5615 (48.15)	871 (62.39)	2970 (57.39)	1774 (34.85)	
Education level (%)	Less than high school	5223 (44.79)	650 (46.56)	2037 (39.36)	2536 (49.82)	< 0.001
	High school graduate or equivalent	3091 (26.51)	320 (22.92)	1408 (27.21)	1363 (26.78)	
	Some college or college graduate	3347 (28.70)	426 (30.52)	1730 (33.43)	1191 (23.40)	
Marital status (%)	Married	6396 (54.85)	892 (63.90)	3138 (60.64)	2366 (46.48)	< 0.001
	Others	5265 (45.15)	504 (36.10)	2037 (39.36)	2724 (53.52)	
Alcohol use (%)	No	5782 (49.58)	481 (34.46)	2236 (43.21)	3065 (60.22)	< 0.001
	Yes	5879 (50.42)	915 (65.54)	2939 (56.79)	2025 (39.78)	
Smoker status (%)	No	8665 (74.31)	1002 (71.78)	3904 (75.44)	3759 (73.85)	0.013
	Yes	2996 (25.69)	394 (28.22)	1271 (24.56)	1331 (26.15)	

**TABLE 2:** Baseline (Wave 12) characteristics of participants for changes in frailty status analyses.

		Overall	Robust	Pre‐frail	Frail	p
*n*	Level	10 178	1539	4028	4611	
Gender (%)	Male	3686 (36.22)	613 (39.83)	1533 (38.06)	1540 (33.40)	< 0.001
	Female	6492 (63.78)	926 (60.17)	2495 (61.94)	3071 (66.60)	
Age (years) (mean [SD])		66.32 (11.55)	61.40 (10.34)	66.03 (10.84)	68.21 (12.02)	< 0.001
Physical activity						
Vigorous (%)	No	8266 (81.21)	1069 (69.46)	3121 (77.48)	4076 (88.40)	< 0.001
	Had	1912 (18.79)	470 (30.54)	907 (22.52)	535 (11.60)	
Moderate (%)	No	6047 (59.41)	655 (42.56)	2116 (52.53)	3276 (71.05)	< 0.001
	Had	4131 (40.59)	884 (57.44)	1912 (47.47)	1335 (28.95)	
Mild (%)	No	5073 (49.84)	553 (35.93)	1711 (42.48)	2809 (60.92)	< 0.001
	Had	5105 (50.16)	986 (64.07)	2317 (57.52)	1802 (39.08)	
Education level (%)	Less than high school	4583 (45.03)	716 (46.52)	1565 (38.85)	2302 (49.92)	< 0.001
	High school graduate or equivalent	2676 (26.29)	331 (21.51)	1099 (27.28)	1246 (27.02)	
	Some college or college graduate	2919 (28.68)	492 (31.97)	1364 (33.86)	1063 (23.05)	
Marital status (%)	Married	5693 (55.93)	996 (64.72)	2510 (62.31)	2187 (47.43)	< 0.001
	Others	4485 (44.07)	543 (35.28)	1518 (37.69)	2424 (52.57)	
Alcohol use (%)	No	4939 (48.53)	537 (34.89)	1694 (42.06)	2708 (58.73)	< 0.001
	Yes	5239 (51.47)	1002 (65.11)	2334 (57.94)	1903 (41.27)	
Smoker status (%)	No	7514 (73.83)	1107 (71.93)	3054 (75.82)	3353 (72.72)	0.001
	Yes	2664 (26.17)	432 (28.07)	974 (24.18)	1258 (27.28)	

### 3.2 Baseline Frailty Status and Cancer Risk

Baseline frailty was significantly associated with subsequent cancer incidence (Table 3). After adjustment for confounding factors, both frail (HR 1.61, 95% CI 1.27–2.02) and prefrail (HR 1.46, 95% CI 1.17–1.83) individuals demonstrated an increased risk of developing cancer compared to their robust counterparts. Among participants with FI > 0.1, the proportion of individuals diagnosed with cancer was higher than that among robust participants (Figure S1).

**TABLE 3:** Association of baseline frailty status with risks of incident cancer diagnoses.

	HRS		
	Events/*n*	HR (95% CI)^a^	*p* ^a^
Robust	95/1396	1 (reference)	
Pre‐frail	566/5171	1.46 (1.17–1.83)	< 0.001
Frail	549/5084	1.61 (1.27–2.02)	< 0.001

Abbreviation: HRS, Health and Retirement Study.

^a^ HR and *p* were adjusted for age, sex, education, marital status, smoking status, drinking status, physical activity and hypertension.

### 3.3 Frailty Transitions and Cancer Risk

We further explored the dynamic nature of frailty by assessing changes over a 2‐year period (Table 4). Among initially robust individuals, 35.2% transitioned to a prefrail or frail state, whereas 16.5% of those who were frail at baseline showed improvement.

**TABLE 4:** Number and percentage of the changes in frailty status.

Baseline	The second survey	*n*	(%)
Robust	Robust	836	65.0%
	Pre‐frail	420	32.6%
	Frail	31	2.4%
Pre‐frail	Robust	673	14.5%
	Pre‐frail	2941	63.4%
	Frail	1025	22.1%
Frail	Robust	34	0.8%
	Pre‐frail	667	15.7%
	Frail	3555	83.5%

As shown in Table 5, transitioning from robustness to a more vulnerable state was linked to a significantly higher risk of incident cancer (HR 2.50, 95% CI 1.74–3.61). Conversely, Frail individuals who improved to a more favourable status (either pre‐frail or robust) experienced a lower cancer risk compared with those who remained frail (HR 0.66, 95% CI 0.48–0.90). Similarly, among prefrail individuals, improvement to robustness was protective (HR 0.51, 95% CI 0.34–0.76), whereas deterioration to frailty was associated with elevated risk.

**TABLE 5:** Association of changes in frailty status with risks of incident cancer diagnoses.

	HRS		
	Events/*n*	HR (95% CI)^a^	*p* ^a^
Robust status in baseline			
Stable robust	54/832	1 (reference)	
Robust to pre‐frail/frail	71/451	2.50 (1.74–3.61)	< 0.001
Pre‐frail status in baseline			
Stable pre‐frail	250/2941	1 (reference)	
Pre‐frail to robust	27/673	0.51 (0.34–0.76)	< 0.001
Pre‐frail to frail	121/1025	1.46 (1.17–1.82)	< 0.001
Frail status in baseline			
Stable frail	316/3555	1 (reference)	
Frail to robust/pre‐frail	46/701	0.66 (0.48–0.90)	0.01

Abbreviation: HRS, Health and Retirement Study.

^a^ HR and *p* were adjusted for age, sex, education, marital status, smoking status, drinking status, physical activity and hypertension.

Regarding factors associated with changes in frailty status, we provided descriptive analyses focusing on physical activity, smoking and alcohol status. The results showed that participants whose frailty status improved were more likely to have increased their physical activity (from inactivity to regular physical activity) compared with those who remained frail. Although the improvements in smoking and alcohol status (from current to non‐use) were relatively modest, participants with improved frailty status still had higher proportions of these positive lifestyle changes than those who remained frail (Table S2).

Increases in FI (∆FI) over time were independently associated with elevated cancer risk. Participants with the greatest frailty progression (upper tertile of ∆FI) were at significantly increased risk compared to those with minimal change (HR 1.35, 95% CI 1.13–1.63; Table 6), also showing a significant trend (*p* for trend = 0.001).

**TABLE 6:** Association of change in FI with risks of incident cancer diagnoses.

	HRS		
	Events/*n*	HR (95% CI)^a^	*p* ^a^
Q1 of △ FI	200/2548	1 (reference)	
Q2 of △ FI	203/2508	0.99 (0.81–1.20)	0.910
Q3 of △ FI	216/2576	1.03 (0.85–1.25)	0.77
Q4 of △ FI	266/2546	1.35 (1.13–1.63)	0.001
*p* for trend test		0.001	

*Note:* △ FI was calculated by the FI at the Wave 12 minus the FI at the Wave 10. Q1 was the lower quartile, and Q4 was the upper quartile.

Abbreviations: FI, frailty index; HRS, Health and Retirement Study.

^a^ HR and *p* were adjusted for age, sex, education, marital status, smoking status, drinking status, physical activity and hypertension.

### 3.4 Sensitivity Analyses

To ensure the robustness of our findings, competing risk analyses were conducted to account for mortality (Tables S3–S5), yielding consistent results. Given the ongoing debate regarding appropriate FI cut‐off values, we reclassified frailty categories using two alternative, commonly applied schemes; associations between changes in frailty status and incident cancer remained consistent under both definitions (Tables S6 and S7). In addition, when a 31‐item FI was constructed by incorporating smoking and alcohol use items, the results were consistent with the main analyses (Tables S8–S10). The observed associations also persisted after further adjustment for baseline FI or changes in frailty status (Tables S11–S13). Moreover, to minimize potential reverse causality, we repeated the main analyses after excluding participants who developed cancer within the first 3 months of follow‐up, and the results remained largely unchanged (Tables S14 and S15). Stratified analyses further demonstrated consistent associations across sex and age groups, with similar findings observed among males and females (Tables S16 and S17 and Figures S2 and S3) as well as middle‐aged and older adults (Tables S19–S21).

## 4 Discussion

In this large prospective cohort study of older adults, we examined the associations between baseline frailty status, changes in frailty status over time and subsequent cancer incidence. Compared with robust participants, those who were prefrail or frail at baseline had a significantly higher risk of cancer events. Notably, robust individuals who progressed to prefrail or frail status during follow‐up were also at elevated risk of developing cancer compared to those who remained robust. In contrast, participants who transitioned from prefrail to robust, or from frail to prefrail or robust status, experienced a lower risk of incident cancer relative to those with persistent frailty. These findings suggest that both frailty status and its longitudinal trajectory are important predictors of future cancer risk.

Frailty is increasingly recognized in oncology, with the frailty construct and several established instruments showing relevance to treatment tolerance, prognosis and patient‐reported outcomes [4, 13, 14]. The specific 29‐item FI used in our study, however, has not been externally validated for cancer outcomes. Prior research has demonstrated that frail patients with cancer are more likely to experience chemotherapy‐related adverse events, postoperative complications and treatment discontinuation [15, 16, 17]. Nevertheless, previous studies have clearly reported that frailty is associated with an increased risk of cancer; these studies have primarily focused on the simple cross‐sectional association between baseline frailty and cancer risk, and our findings are consistent with theirs [16]. Additionally, frailty has been associated with increased mortality and poorer quality of life among cancer survivors [18, 19, 20]. These associations highlight the clinical significance of frailty not only as a marker of vulnerability but also as a potential target for supportive interventions aimed at optimizing cancer care in older populations.

Despite the growing literature on frailty and cancer outcomes, few studies have examined the role of dynamic changes in frailty status in relation to cancer development. Whereas most oncology studies have assessed frailty at a single time point, typically at diagnosis or the initiation of treatment [8, 21, 22]. In contrast, only a smaller body of work has examined antecedent frailty in community or pre‐diagnostic settings and explored its relationship with subsequent cancer risk and outcomes [16]. By incorporating longitudinal assessments of frailty and analysing transitions between frailty states, our study adds to the emerging evidence that changes in frailty status over time may provide important prognostic information. The observed associations between frailty progression and increased cancer risk, as well as between frailty improvement and reduced risk, underscore the relevance of frailty as a dynamic and potentially modifiable factor in cancer prevention.

Our findings support the importance of routinely monitoring frailty trajectories in ageing populations. The ability to identify individuals who are progressing towards or recovering from frailty may offer novel opportunities for early intervention and risk stratification. In particular, interventions targeting frailty progression—such as nutritional supplementation, exercise programmes or comprehensive geriatric assessment—may have potential implications for cancer prevention strategies among older adults. Future studies are warranted to explore whether reversing or slowing frailty progression translates into lower cancer risk across diverse populations and cancer types.

A major strength of our study lies in its prospective design, large sample size and repeated frailty assessments over time, which enabled us to capture detailed trajectories of frailty status and examine their associations with incident cancer risk. Furthermore, the use of a validated frailty index and adjustment for a wide range of potential confounders enhance the robustness of our findings.

However, several limitations should be acknowledged. First, although we adjusted for many known confounders, residual confounding by unmeasured factors—such as subclinical inflammation genetic predisposition, and other biological factors—cannot be excluded. Second, although frailty transitions were assessed over a defined period, it remains possible that some changes in frailty status reflected early manifestations of undiagnosed cancer. Future studies with longer follow‐up and lag‐time analyses are needed to clarify the directionality of these associations.

## 5 Conclusion

Dynamic changes in frailty status were significantly associated with subsequent cancer risk. Individuals experiencing frailty progression had an elevated risk of incident cancer, whereas those who recovered from frailty showed a reduced risk. These findings highlight the importance of early identification and management of frailty transitions, which may help inform preventive strategies in ageing populations. Future studies are warranted to explore whether targeted strategies aimed at preventing or reversing frailty could contribute to cancer risk reduction among ageing populations.

## Funding

This work was supported by the National Key Research and Development Program (2022YFC2009600 and 2022YFC2009601), Laboratory for Clinical Medicine, Capital Medical University (2023‐SYJCLC02) and National Multidisciplinary Cooperative Diagnosis and Treatment Capacity Project for Major Diseases: Comprehensive Treatment and Management of Critically Ill Elderly Inpatients (2019. YLFW).

## Conflicts of Interest

The authors declare no conflicts of interest.

## Supporting information


**Figure S1:** Incidence rate of cancer dependent on baseline FI.
**Figure S2:** Association of baseline frailty status with risks of incident cancer diagnoses in stratified analyses.
**Figure S3:** Association of changes in frailty status with risks of incident cancer diagnoses in stratified analyses.

**Table S1:** The 29 items used to construct the frailty index.
**Table S2:** Behavioural changes across changes in frailty status.
**Table S3:** Association of baseline frailty status with risks of incident cancer diagnoses.
**Table S4:** Association of changes in frailty status with risks of incident cancer diagnoses.
**Table S5:** Association of change in FI with risks of incident cancer diagnoses.
**Table S6:** Association of baseline frailty status with risks of incident cancer diagnoses stratified when using another two cut‐off values of FI.
**Table S7:** Association of changes in frailty status with risks of incident cancer diagnoses stratified when using another two cut‐off values.
**Table S8:** Association of baseline frailty status with risks of incident cancer diagnoses by 31‐item FI.
**Table S9:** Association of changes in frailty status with risks of incident cancer diagnoses by 31‐item FI.
**Table S10:** Association of change in FI with risks of incident cancer diagnoses by 31‐item FI.
**Table S11:** Association of baseline frailty status with risks of incident cancer diagnoses after further adjusting for the changes in frailty status.
**Table S12:** Association of changes in frailty status with risks of incident cancer diagnoses after further adjusting for the baseline FI.
**Table S13:** Association of change in FI with risks of incident cancer diagnoses after further adjusting for the baseline FI.
**Table S14:** Association of changes in frailty status with risks of incident cancer diagnoses after excluding participants with incident cancer during the first 3 months of follow‐up.
**Table S15:** Association of change in FI with risks of incident cancer diagnoses after excluding participants with incident cancer during the first 3 months of follow‐up.
**Table S16:** Association of baseline frailty status with risks of incident cancer diagnoses stratified by sex.
**Table S17:** Association of changes in frailty status with risks of incident cancer diagnoses stratified by sex.
**Table S18:** Association of change in FI with risks of incident cancer diagnoses stratified by sex.
**Table S19:** Association of baseline frailty status with risks of incident cancer diagnoses stratified by age.
**Table S20:** Association of changes in frailty status with risks of incident cancer diagnoses stratified by age.
**Table S21:** Association of change in FI with risks of incident cancer diagnoses stratified by age.

## Data Availability

The datasets generated during and/or analysed during the current study are available from the corresponding author on reasonable request.
