# Supplementary material for: Changes in Frailty and Incident Cancer: Evidence From the Health and Retirement Study
Source: J Cachexia Sarcopenia Muscle. 2025 Dec 16;16(6):e70164. doi: 10.1002/jcsm.70164 (PMC12707304; doi:10.1002/jcsm.70164)
Supplement: Supplementary file 1 — Figure S1: Incidence rate of cancer dependent on baseline FI. Figure S2: Association of baseline frailty status with risks of incident cancer diagnoses in stratified analyses. Figure S3: Association of changes in frailty status with risks of incident cancer diagnoses in stratified analyses. [file JCSM-16-e70164-s001.docx]

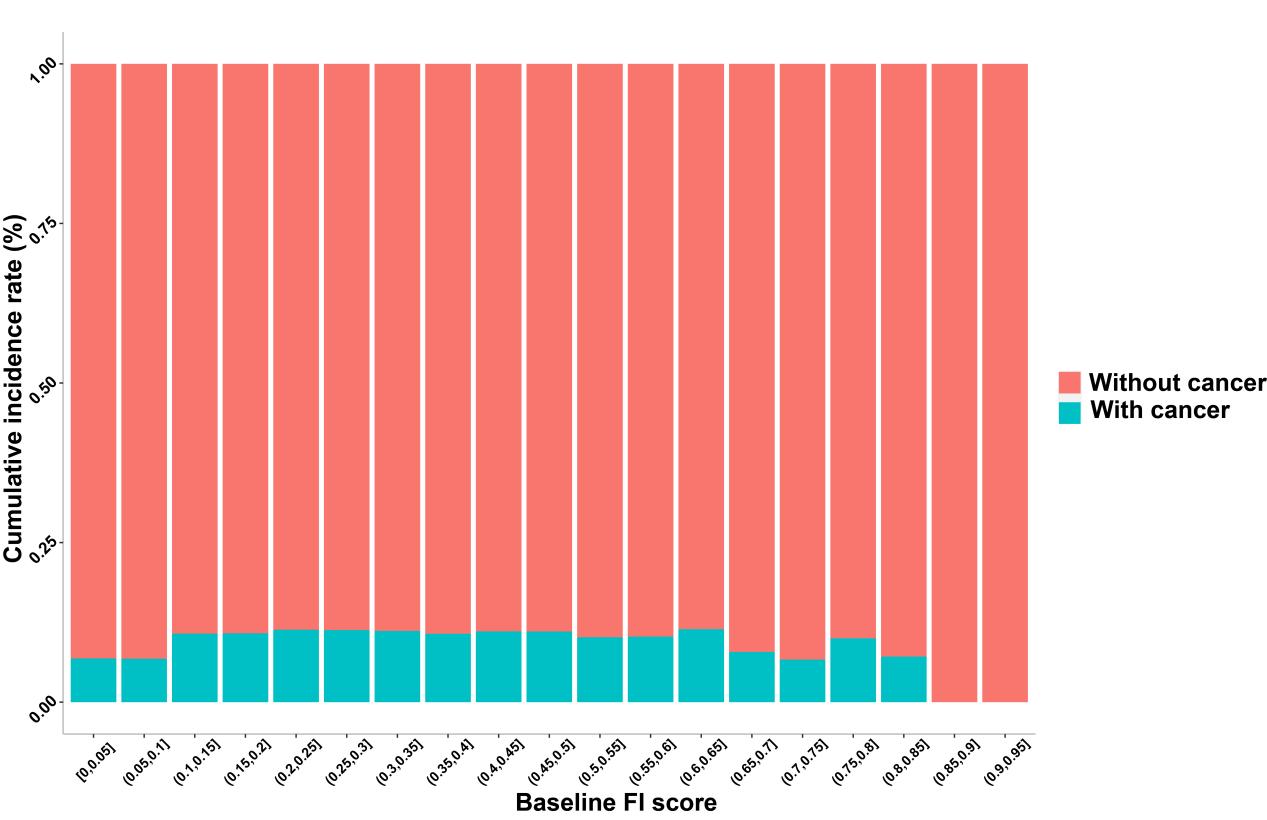


**Figure S1.** Incidence rate of cancer dependent on baseline FI


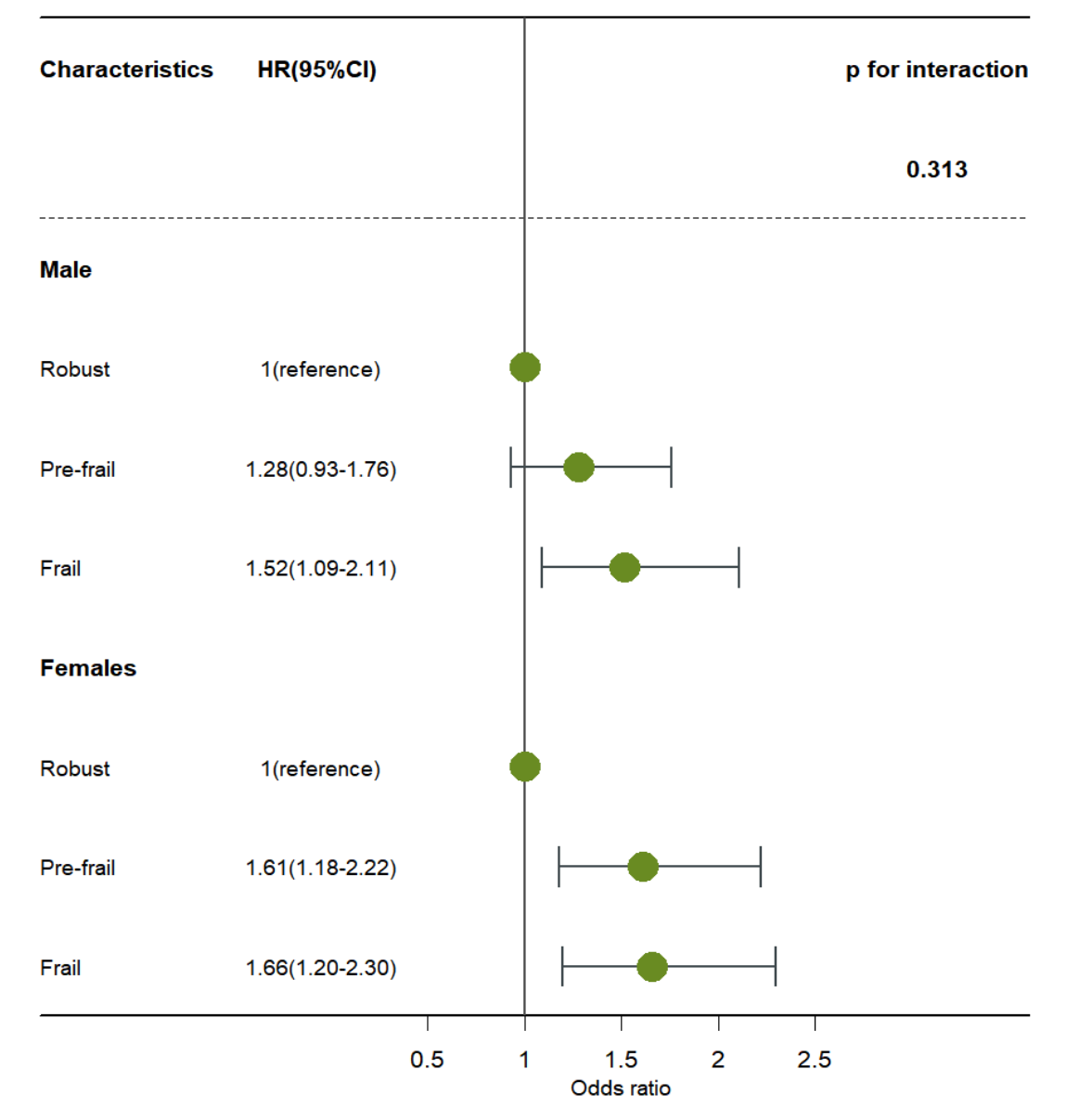


**Figure S2.** Association of baseline frailty status with risks of incident cancer diagnoses in stratified analyses.


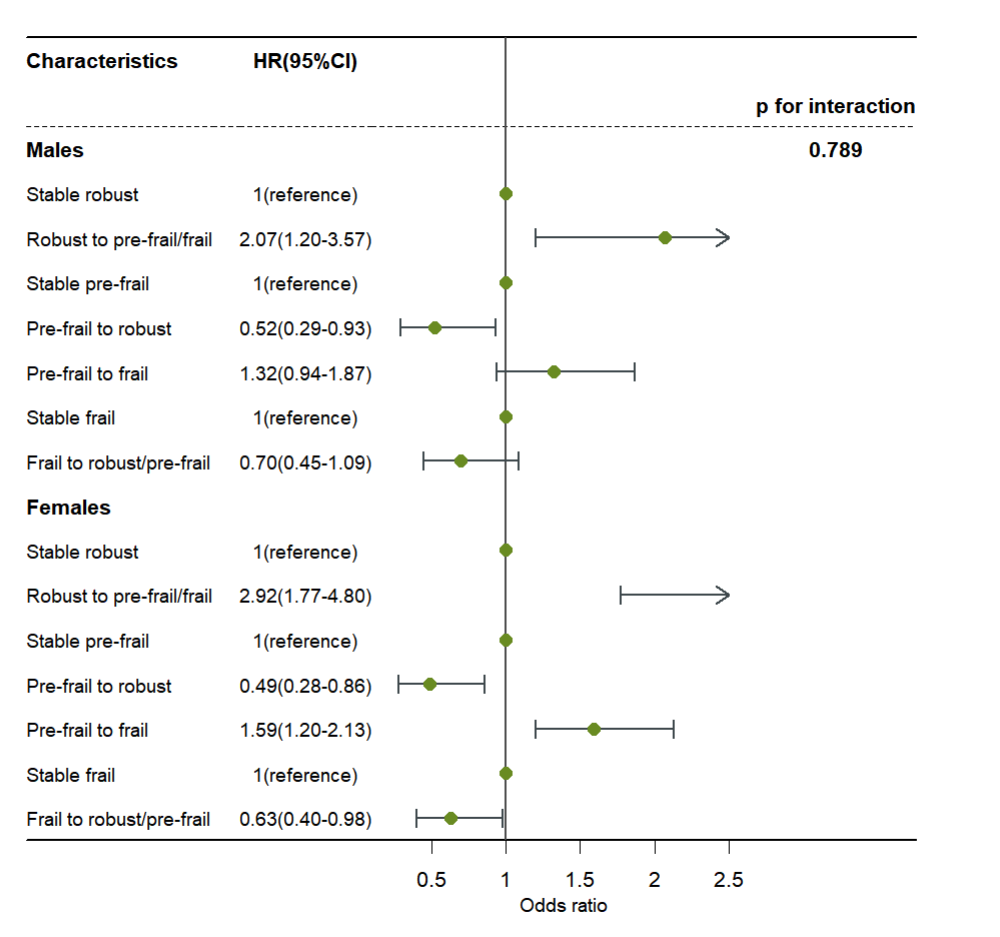


**Figure S3.** Association of changes in frailty status with risks of incident cancer diagnoses in stratified analyses.
